# Effectiveness of a Nurse-Led Mobile-Based Health Coaching Program for Patients With Prostate Cancer at High Risk of Metabolic Syndrome: Randomized Waitlist Controlled Trial

**DOI:** 10.2196/47102

**Published:** 2024-02-01

**Authors:** Kyoungjin Lee, Jeongok Park, Eui Geum Oh, JuHee Lee, Chang Park, Young Deuk Choi

**Affiliations:** 1 College of Nursing and Brain Korea 21 FOUR Project Yonsei University Seoul Republic of Korea; 2 College of Nursing Kyungbok University Namyangju Republic of Korea; 3 Mo-Im Kim Nursing Research Institute, College of Nursing Yonsei University Seoul Republic of Korea; 4 Department of Population Health Nursing Science, College of Nursing University of Illinois at Chicago Chicago, IL United States; 5 College of Medicine Yonsei University Seoul Republic of Korea

**Keywords:** nurses, prostate neoplasms, healthy lifestyle, metabolic syndrome, exercise, diet, mobile phone

## Abstract

**Background:**

Androgen deprivation therapy (ADT), a standard treatment for prostate cancer (PC), causes many physical side effects. In particular, it causes metabolic changes such as fasting glucose abnormalities or accumulation of body fat, and its continuation can lead to metabolic syndrome (MetS), which is closely related to diabetes and cardiovascular disease. Therefore, it is important to maintain and practice a healthy lifestyle in patients with PC.

**Objective:**

This study aims to evaluate the effectiveness of a nurse-led mobile-based program that aims to promote a healthy lifestyle in patients with PC undergoing ADT with MetS risk factors.

**Methods:**

This was a single-blind, randomized, waitlist control interventional study. A total of 48 patients were randomly assigned to the experimental and waitlist control groups at the urology cancer clinic of a tertiary general hospital in South Korea. The inclusion criteria were patients who had undergone ADT for >6 months, had at least 1 of the 5 MetS components in the abnormal range, and could access a mobile-based education program. The experimental group attended a 4-week mobile-based program on exercise and diet that included counseling and encouragement to maintain a healthy lifestyle, whereas the control group was placed on a waitlist and received usual care during the follow-up period, followed by the intervention. The primary outcome was a change in the lifestyle score. The secondary outcomes were changes in 5 MetS components, body composition, and health-related quality of life. The outcomes were measured at 6 weeks and 12 weeks after the initiation of the intervention. Each participant was assigned to each group in a sequential order of enrollment in a 4×4 permuted block design randomization table generated in the SAS (SAS Institute) statistical program. A linear mixed model was used for statistical analysis.

**Results:**

A total of 24 participants were randomly assigned to each group; however, 2 participants in the experimental group dropped out for personal reasons before starting the intervention. Finally, 46 participants were included in the intention-to-treat analysis. The experimental group showed more positive changes in the healthy lifestyle score (β=29.23; *P*≤.001), level of each MetS component (fasting blood sugar: β=−12.0; *P*=.05 and abdominal circumference: β=−2.49; *P*=.049), body composition (body weight: β=−1.52; *P*<.001 and BMI: β=−0.55; *P*<.001), and the urinary irritative and obstructive domain of health-related quality of life (β=14.63; *P*<.001) over time than the waitlist control group.

**Conclusions:**

Lifestyle changes through nurse-led education can improve level of each MetS components, body composition, and ADT side effects. Nurses can induce positive changes in patients’ lifestyles and improve the self-management of patients starting ADT through this program.

**Trial Registration:**

Clinical Research Information Service KCT0006560; http://tinyurl.com/yhvj4vwh

## Introduction

### Adverse Effects of Androgen Deprivation Therapy in Prostate Cancer

Prostate cancer (PC) is a commonly occurring cancer in men worldwide, and it is the fourth most common cancer among adult men in South Korea [1]. The incidence of PC in South Korea has increased rapidly in recent years, from 2.2% in 2000 to 14.3% in 2018 [1], owing to changes in dietary patterns and the development of new diagnostic technologies. PC is affected by androgenic activity in the body [2]. Therefore, the goal of treatment is to either remove androgens using castration or neutralize the effects of androgens. Androgen deprivation therapy (ADT), which disrupts the mechanisms that create androgens, is a standard treatment for PC [3]. Initially, ADT was used to treat patients with metastatic PC or older persons with cancer with limited treatment options. However, in recent years, there has been a gradual expansion in the use of ADT to include treating patients in the early stages of PC [4,5].

Repeated ADT can lead to climacteric symptoms in male individuals [6], including sexual dysfunction, hot flashes [7], gynecomastia [8], depression, fatigue, changes in sleep patterns [9], loss of muscle strength [10,11], osteoporosis [12], metabolic syndrome (MetS) [13], and cardiovascular disease [14,15]. These issues can reduce the quality of life of patients and may even be life threatening [16,17]. Specifically, evidence suggests that ADT leads to metabolic changes [13,18]. MetS, also called insulin resistance syndrome, is the clustering of several risk factors associated with obesity. It is closely related to diabetes [14,15,19] and has also been identified as a major cause of cardiovascular disease owing to its association with dyslipidemia, diabetes, and hypertension [14,15]. PC and MetS have a close relationship: patients with PC who received ADT were found to be 2.5 times more likely to have MetS than those who did not receive ADT [18]. A cross-sectional analysis using Korean National Health Insurance Service data found that the prevalence rate of MetS in patients with PC was 40.1%, which is higher than the 34.5% prevalence rate in older Korean male adults [20]. In addition, as age increases, MetS has been found to increase by 8 times in patients with PC [21]. Given that most patients with PC are older adults, ADT-induced metabolic problems coupled with aging can lead to deterioration of health, resulting in cardiovascular disease or death [22]. Despite the necessity of severe side effects management for ADT, >50% of patients with PC are unaware of these problems [23], and most health care providers tend to focus on cancer treatment. Moreover, no protocols have been developed to manage ADT-induced MetS in patients with PC [24,25].

### Importance of Improving Self-Management Skill for ADT-Induced MetS

ADT-induced MetS causes metabolic changes that are different from those caused by classic MetS including changes in body composition [26]. Androgens are a group of male sex hormones, and androgen level reduction leads to an increase in body fat percentage and a decrease in fat-free mass, such as muscle loss and decreased bone density. Although classic MetS is characterized by an increase in visceral fat, there is insufficient evidence to determine whether or not this also occurs in patients with PC [27,28]. Implementing a classic MetS management program is not suitable for patients with PC because ADT-induced MetS has different characteristics than classic MetS, and each patient experiences different side effects, including physical and emotional problems. For example, 80% of patients with PC experience hot flashes and 12% to 14% experience breast tenderness and depression [29].

MetS is a representative chronic lifestyle disease that requires individual self-management. The World Health Organization emphasizes that lifestyle is the most critical factor affecting health status [30]. The current health care system appears to focus its attention and investments toward the discovery and treatment of the causes of disease rather than lifestyle. However, lifestyle modification is the most effective way to reduce the incidence of cardiovascular complications caused by MetS [31]. In general, lifestyle modifications, including exercise and a nutritional diet, are known to result in lower occurrence rates of MetS and a lower risk of cardiovascular disease [31]. Such lifestyle modifications require improving self-management skills. Self-management is a lifelong task that requires engagement in activities that promote good health [32]. Improving self-management allows patients to maintain active lives, leading to a better quality of life [32].

Although the optimal duration of ADT remains undefined, patients with PC typically receive ADT for 2 to 3 years [33]. Furthermore, as metabolic changes begin at least 3 months after ADT initiation, it is very important for patients with PCs to improve their self-management skills from the beginning of ADT. In addition, health care providers should be aware of ADT-related side effects from an early stage and mediate metabolic changes of PC through education. Therefore, newly developed health coaching programs for patients with PC should be configured differently from previous iterations [34-36]. In other words, the focus should be on improving self-management skills to encourage lifestyle changes that take into consideration the side effects of ADT from the beginning of ADT administration. However, effective self-management can be challenging to maintain. In addition, the COVID-19 pandemic has diminished physical activity and nutritional quality worldwide [37]. The World Health Organization has emphasized the importance of exercise and maintaining a healthy lifestyle during the pandemic [38]. Web-based education has relatively few limitations in terms of time and space, and it has the advantage of being able to feature various types of media and teaching and learning materials compared with offline education programs [39].

### Aims

This study aimed to evaluate the effectiveness of a nurse-led mobile-based health coaching program that promotes healthy lifestyle changes, normal range of MetS components, and health-related quality of life (HRQoL) for patients with PC receiving ADT. The primary aim was to identify the changes in (1) healthy lifestyle through this program, and the secondary aims were to identify the changes in (1) the levels of each MetS component, including blood pressure, fasting blood sugar (FBS), high-density lipoprotein (HDL) cholesterol, triglyceride, and abdominal circumference (AC); (2) body composition, such as body weight, BMI, skeletal muscle mass, fat mass, and fat percentage; and (3) quality of life.

## Methods

### Participants

The study population consisted of men who were diagnosed with PC at the urology cancer center of a single tertiary general hospital in South Korea. The inclusion criteria were (1) patients with PC who had been receiving ADT for >6 months at the time of enrollment in this study, (2) those with an abnormal range of at least 1 of the 5 MetS components, (3) those who were fully aware of the object and contents of the study and voluntarily participated, (4) those who understood spoken and written Korean and who could communicate without cognitive impairment, and (5) those who had a smartphone and were able to access the mobile-based education program. The exclusion criteria were (1) patients diagnosed with and treated for cancers other than PC; (2) those who had undergone surgery or chemotherapy for <3 months before the start of the study; (3) those who answered “yes” to ≥1 question of the Physical Activity Readiness Questionnaires; (4) those with cardiovascular diseases such as unstable angina pectoris, uncontrolled blood pressure, myocardial infarction, or comorbidities such as a musculoskeletal or nervous system disease; (5) those who were participating in other research programs; (6) those who had a change in medication to control their blood pressure, cholesterol, or blood sugar <3 months before the start of the study; and (7) those who had difficulty with typical daily activities.

### Ethical Considerations

This study was approved by the Institutional Review Board of the Severance Hospital, Yonsei University Health System (4-2020-0889). We informed all participants of the purpose of this study, process, methods, voluntary participation terms, and the possible risks and benefits of participation. Furthermore, we gave the participants 1 week to consider their participation in the study before deciding whether to sign the informed consent form. All study participants were compensated with KRW 50,000 (approximately US $43) as a gift at both the beginning and end of the study.

### Enrollment Process

**Figure 1:**
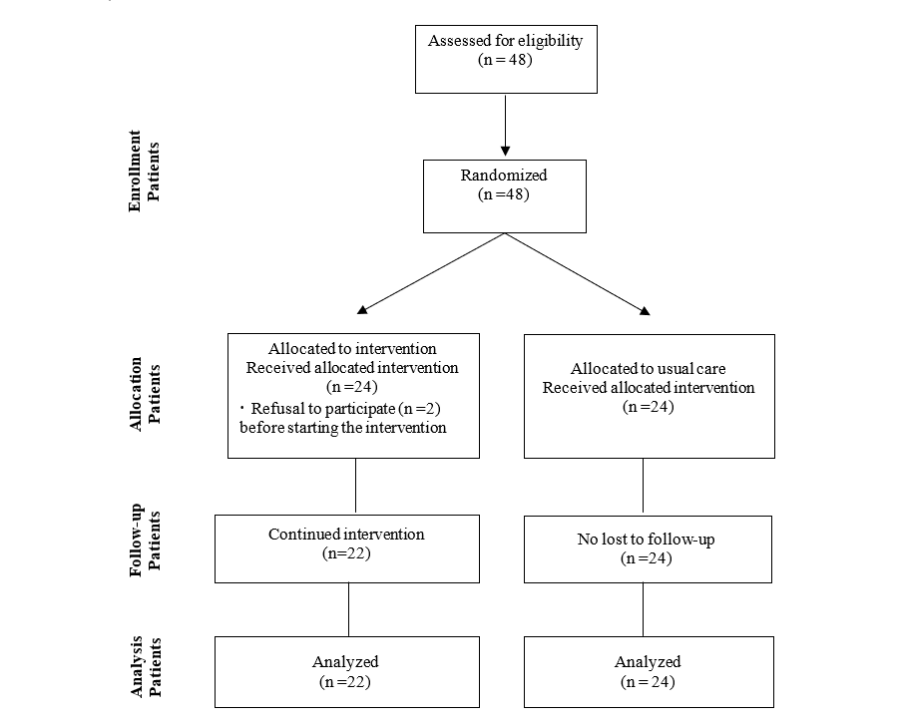
Modified CONSORT (Consolidated Standards of Reporting Trials) flow diagram for individual randomized controlled trials.

To recruit participants, we posted recruitment announcements on the bulletin board of a urology cancer center. On the day of outpatient visits, we met face-to-face with patients who indicated an interest in participating in the study. We provided potential participants with the opportune time to ask questions. Then, we fully informed the potential participants about all facets of the study and invited them to voluntarily sign the informed consent form. Finally, we registered the participants after screening for the inclusion and exclusion criteria (Figure 1). The allocation process for this study was conducted jointly with a research assistant to ensure that all enrollments were transparent.

Using *F* test in the G*Power program version 3.1 (Heinrich Heine University) [40], we calculated the sample size to identify the variance difference of repeated data between the 2 groups. The minimum number of participants was calculated to be 41 based on an effect size of 0.51, which was derived from the study by Bourke et al [41], with a significance level of .05, a power of 0.08, and 3 repeat measures. Considering a projected dropout rate of 15% [41], the total sample size was set at 48. Therefore, 24 participants were registered in each group. We assigned the participants randomly into experimental and waitlist control groups using a pregenerated sequence in SAS 9.4 (SAS Institute) based on the 2×2 permuted randomized block method. Using this method, 4 people are grouped as 1 block, and the 1 block is then divided into 2 groups. Then, we assigned the groups at a 1:1 ratio. The participants did not know which group they were assigned to. They received the intervention individually, which minimized the risk of contamination between groups.

### Research Design and Setting

This was a single-blind, single-center randomized waitlist controlled trial. It used a pre- and posttest design and a 2×2 permuted randomization block method. We prepared an allocation table for each group before participants were enrolled. Each participant was assigned to an experimental or a waitlist control group in the order of enrollment using a 2×2 randomization table generated in the SAS program (SAS Institute). The protocol for this study was registered with the Clinical Research Information Service (registration no. KCT0006560). We recruited the participants from a tertiary general hospital located in Seoul, South Korea.

### Intervention

To improve the positive health behaviors of patients with PC receiving ADT, a nurse-led mobile-based health coaching program based on the analyze, design, develop, implement, and evaluate model and the information-motivation-behavioral (IMB) skills model was developed. The analyze, design, develop, implement, and evaluate model, known as an instructional design model, is a representative framework used by teaching and learning methods. The IMB model has been widely used as a theoretical basis for developing interventions that aim to encourage patients with chronic diseases to maintain positive health behaviors. According to the IMB model, acquiring sufficient information, enhancing motivation, acquiring the skills required to perform a behavior, and improving self-efficacy are the factors that lead to long-term behavioral changes that improve subjective and objective health outcomes [42]. The nurse-led mobile-based health coaching program used an individualized approach to ensure that the IMB skills addressed in the program were best suited to each patient to improve their specific lifestyle patterns. In this program, self-management information included personalized recommendations for diet and exercise and strategies to manage both individual ADT-induced side effects and common side effects from other prescribed drugs. Motivation strategies included health contracting, individual goal setting, encouragement and continuous counseling, and rewarding. In addition, we encouraged the patients’ main caregivers to become involved in their patients’ behavioral self-management. Behavioral self-management skills included sharing vicarious experiences (eg, sharing success stories), self-monitoring through exercises, and maintaining a nutrition diary.

**Figure 2:**
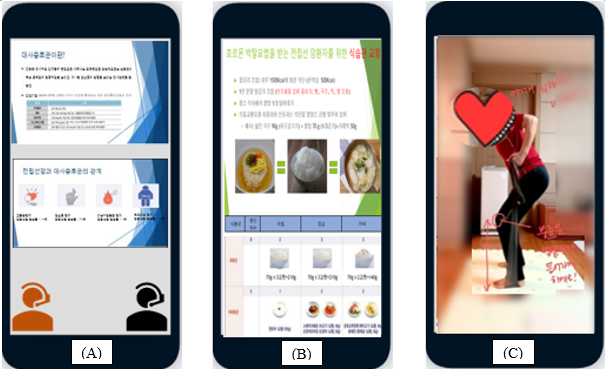
Educational material: (A) education on information and management of metabolic syndrome via the internet using Zoom (Zoom Video Communications, Inc), (B) personalized diet coaching, and (C) personalized exercise coaching: providing feedback describing the correct motion.

The program consisted of a 4-week intensive program conducted via one-on-one Zoom (Zoom Video Communication, Inc) meetings, followed by an 8-week maintenance program conducted via individual contact through telephone calls and KakaoTalk (Kakao Corporation), which is a popular Korean SMS text messaging app (Figure 2). Participants were provided with relevant information and a to-do list regarding exercise and diet once a week for 4 weeks in an intensive program. We provided an educational package that included a booklet containing PowerPoint (Microsoft Corporation) slides, exercise and nutritional diaries, exercise video files, TheraBand resistance bands (THERABAND), and a pedometer. We focused on overcoming barriers to healthy diet and exercise. For example, we recommended alternative exercises for participants with knee pain, which included a higher proportion of movements performed in a sitting position. Furthermore, the participants received feedback to address problems at the end of every web-based meeting during the intensive program and via SMS text messages and telephone calls during the maintenance program. When participants felt uncertain about an exercise motion, they recorded a video of themselves performing the exercise and sent it to us. We then provided feedback describing the correct motion, using the video as a reference. Regarding diet, we focused on changing unhealthy eating habits, controlling participants’ daily calorie intake, encouraging a low-fat and low-carbohydrate diet, and ensuring adequate protein intake. Participants were educated on the proportions of food from each food group they required per day according to their individual daily recommended calories and the diabetic exchange diet. Feedback and questions related to the diet program were addressed via KakaoTalk. We recommended implementing the following strategies for maintaining healthy behaviors and enhancing self-efficacy once a week or more during the maintenance period: vicarious experiences (eg, sharing success stories), emotional support, encouragement, keeping an exercise and nutrition diary, and goal reminders. To minimize the expected bias that can occur in an intervention study, we conducted the intervention according to the study protocol and used a checklist to ensure consistency between the experimental and waitlist control groups. The control group was placed on a waitlist and only received usual care during the intervention period. After completing data collection, we provided them with the same mobile-based program and educational materials as the experimental group (time point 3; T3). A group of experts evaluated this program to confirm its content and construct validity.

### Data Collection

Data collection happened at 3 different time points: baseline (time point 1; T1), 6 weeks after the beginning of the intervention (time point 2; T2), and 12 weeks after the beginning of the intervention (T3). We collected T1 and T3 data on a face-to-face basis on the day the patient visited the urology cancer center. Variables that required physical contact, such as AC and grip strength, were measured together using a web-based survey at T1 and T3 but not at T2. At T2, we collected data collection on variables, including lifestyle score and HRQoL, on a non–face-to-face basis using a web-based survey. Clinical data that were obtained included treatment type, ADT exposure time, cancer stage, biochemical data, and Gleason score from electronic medical records (EMRs) at T1.

### Outcomes

#### Lifestyle Score

Lifestyle score was the primary outcome of this study. Lifestyle changes were evaluated using the lifestyle evaluation tool by Kang [43] to assess the health behaviors of patients with MetS. A higher score indicates better self-management behaviors. This assessment consists of 36 items across 6 domains: physical activity and weight control, dietary habits, drinking and smoking, sleep and rest, stress, and drug and health management [43].

We recorded responses on a 4-point scale with the options *not at all*, *sometimes*, *often,* and *always*. Total scores could range from 36 to 144 points, with a higher score indicating a healthier lifestyle. The Cronbach α of the original study was .92 (physical activity and weight control=.87, dietary habits=.87, drinking and smoking=.87, sleep and rest=.86, stress=.74, and drug and health management=.70). In this study, Cronbach α was .82 (physical activity and weight control=.90, dietary habits=.86, drinking and smoking=.41, sleep and rest=.67, stress=.63, and drug and health management=.54).

#### MetS Components

The components of MetS consist of FBS, AC, blood pressure (both systolic and diastolic), fasting triglyceride level, and fasting HDL cholesterol. We instructed the participants not to have a meal and not to take antihypertensive drugs on the test day. Then, at the urology cancer center, we first measured AC using a tape at the umbilicus between the highest point of the iliac crest and the lower edge of the 12th rib with an error range of 0.5 cm while the participants fasted. Second, we measured the blood pressure twice on the participant’s nondominant arm using a TM-2657P device (A&D Company Limited) after they had rested for at least 10 minutes. The average systolic blood pressure (SBP) and diastolic blood pressure (DBP) were calculated.

#### Biochemical Data

Biochemical data included the levels of the following MetS components: fasting triglyceride, fasting HDL cholesterol, and FBS. These variables were measured using blood samples collected while participants had fasted for >6 hours, and the data were obtained from EMRs.

#### Body Composition

Body composition data were obtained using a body composition analyzer (InBody H20B [Biospace]). We instructed the participants to stand upright and hold the handle attached to the measurement device, which put them into contact with 8 electrodes (2 each on both hands and both feet). Body composition measurements included height (cm), body weight (kg), body fat mass (kg), body fat percentage (%), skeletal muscle mass (kg), and BMI (kg/m^2^).

#### HRQoL Measurement Tool

We measured HRQoL using the Korean version of the 26-item Expanded Prostate Cancer Index Composite (EPIC-26). EPIC-26 is a short-form version of the original expanded PC index composite (EPIC) tool, which contains 50 items. The EPIC tool was developed to understand treatment-related symptoms with a higher degree of sensitivity than previous diagnostic tools and the impact of PC treatment on patients’ lives [44]. Higher scores indicated a better HRQoL, with possible scores ranging from 0 to 100. EPIC-26 consists of 5 symptom domains: urinary incontinence, urinary irritation and obstruction, sexual, bowel, and hormonal. There is no Korean version of EPIC-26, but there is a Korean version of the original 50-item EPIC tool. Therefore, the 26 items from EPIC-26 were extracted from the Korean version of the original 50-item EPIC tool [45], and the survey was conducted using this tool. Permission to use both EPIC-26 and the original 50-item EPIC was granted by the original author.

The Cronbach α of EPIC-26 ranged from .70 to .90 in all domains except for the hormonal domain (Cronbach α=.62). The Cronbach α of the Korean version of EPIC was .83 [45]. The Cronbach α of the tool used in this study was .63 (urinary incontinence=.88, urinary irritation and obstruction=.64, sexual=.84, bowel=.13, and vitality or hormonal=.46).

#### Clinical Data

Disease-related patient information, treatment type, ADT exposure time, cancer stage, and Gleason score were obtained from EMRs.

### Data Analysis

Demographic data, disease-related characteristics, and main outcome variables were analyzed using mean, SD, frequency, and percentage. An independent 1-tailed *t* test and a chi-square test were performed to identify differences between the groups. The equality of variance was assessed before using the pooled variance estimator for the *t* test. Fisher exact test was performed as appropriate. According to the International Conference on Harmonization E9 guideline [46], which provides guidance on statistical principles for clinical trials, a modified intention-to-treat analysis was conducted. As 2 participants withdrew from the study for personal reasons before initiating the intervention, there was a lack of data that could evaluate the effect of the main outcomes. We judged that there would be no difference in the intention-to-treat analysis owing to the low dropout rate (2/48, 4%) and high compliance rate (22/22, 81%). Hypothesis testing was conducted using a 1-tailed test and the PROC MIXED procedure in SAS (SAS Institute). SE estimates were obtained as a result of the PROC MIXED procedure using the *empirical option* to adjust for skewed data from potentially different covariance structures. This method is based on the sandwich estimation approach [47]. It improves variance and covariance with robust and consistent estimates, irrespective of the covariance structure in the actual data. As a follow-up analysis, we determined statistically significant time points within the groups by calculating the difference in the least square means from the baseline at each time point.

## Results

### Overview

The data for this study were collected across 7 months, from March 24 to September 15, 2021. We took approximately 30 minutes per participant to complete data collection. A total of 48 participants were recruited. Two participants in the experimental group declined to participate in the program before starting the intervention, citing personal reasons. A total of 46 participants were finally included in the analysis, with an attrition rate of 4% (2/48).

### Results of General and Disease-Related Characteristics

The general and disease-related characteristics are presented in Table 1. The mean age of the participants was 68.83 (SD 7.09) years. Most participants (41/46, 89%) lived with their spouses or families. Approximately 46% (21/46) of the participants were unemployed, and 35% (16/46) had jobs requiring relatively less physical activity, such as office workers, taxi drivers, and service workers. Most participants (27/46, 59%) were exsmokers, and the mean smoking duration was 16.13 (SD 21.38) pack-years.

**Table 1:** Homogeneity tests in general characteristics, disease-related characteristics, and main outcome variables between groups (N=46).

Variables				Total		Experimental group (n=22)		Control group (n=22)		*χ*^2^ (*df*)		*t* (*df*)		*P* value	
**General characteristics**															
	Age (y), mean (SD)			68.83 (7.09)		67.59 (6.59)		69.96 (7.48)		N/A^a^		−1.13 (44)		.26	
	Monthly income (US $), mean (SD)			3679.77 (4603.72)		4813.0 (6188.7)		2640.9 (2035.5)		N/A		1.57 (44)		.13	
	**Religion, n (%)**														.99
		No	21 (46)		10 (22)		11 (24)		0.0 (1)		N/A				
		Yes	25 (54)		12 (26)		13 (28)		N/A		N/A				
	**Education, n (%)**														.40
		Less than or equal to middle school	27 (59)		11 (24)		16 (35)		0.7 (1)		N/A				
		Greater than or equal to college	19 (41)		11 (24)		8 (17)		N/A		N/A				
	**Living, n (%)**														.77^b^
		Alone	5 (11)		3 (7)		2 (4)		0.5 (2)		N/A				
		With spouse only	21 (46)		9 (20)		12 (26)		N/A		N/A				
		With their family	20 (43)		10 (22)		10 (22)		N/A		N/A				
	**Job-related physical activities, n (%)**														.93^b^
		Unemployed	21 (46)		10 (22)		11 (24)		0.3 (2)		N/A				
		Less active	16 (35)		7 (15)		9 (35)		N/A		N/A				
		Highly active	9 (20)		5 (11)		4 (9)		N/A		N/A				
	**Smoking history**														
		1 pack-year, mean (SD)	16.13 (21.38)		15.78 (21.58)		16.44 (21.65)		N/A		−0.1 (44)		.92		
		Nonsmoker, n (%)	16 (35)		6 (13)		10 (22)		1.6 (2)		N/A		.45^b^		
		Exsmoker, n (%)	27 (59)		15 (33)		12 (26)		N/A		N/A		N/A		
		Current smoker, n (%)	3 (7)		1 (2)		2 (4)		N/A		N/A		N/A		
**Disease characteristics**															
	**Number of comorbidities, mean (SD)**			1.61 (1.04)		1.64 (1.05)		1.58 (2.03)		N/A		0.17 (44)		.87	
		0, n (%)	6 (13)		3 (7)		3 (7)		0.8 (3)		N/A		.87^b^		
		1, n (%)	17 (37)		7 (15)		10 (22)		N/A		N/A		N/A		
		2, n (%)	14 (30)		8 (17)		6 (13)		N/A		N/A		N/A		
		≥3, n (%)	9 (20)		4 (9)		5 (11)		N/A		N/A		N/A		
	**Treatment type, n (%)**														.10
		Operation	16 (35)		5 (11)		11 (24)		2.7 (1)		N/A				
		Operation+radiation	30 (65)		17 (37)		13 (28)		N/A		N/A				
	**ADT^c^ type, n (%)**														
		Antiandrogen	30 (65)		14 (30)		16 (35)		0.0 (1)		N/A		.99		
		Antiandrogen+LHRH^d^	16 (35)		8 (17)		8 (17)		N/A		N/A		N/A		
	ADT duration (month), mean (SD)			40.63 (24.71)		40.64 (22.61)		40.63 (26.98)		N/A		0.0 (44)		.99	
	PSA^e^, mean (SD)			0.07 (0.19)		0.09 (0.27)		0.05 (0.08)		N/A		0.59 (44)		.56	
	Gleason score, mean (SD)			7.80 (0.98)		8.00 (1.07)		7.63 (0.88)		N/A		1.31 (44)		.20	
**Main outcomes**															
	**Lifestyle score^f^, mean (SD)**														
		Total score	99.07 (13.56)		100.50 (12.53)		97.71 (14.57)		N/A		0.70 (44)		.48		
		Exercise and weight loss	19.67 (5.88)		20.23 (5.45)		19.17 (6.32)		N/A		0.61 (44)		.55		
		Diet	42.17 (6.72)		42.77 (6.05)		41.63 (7.38)		N/A		0.57 (44)		.57		
		Alcohol and smoking	9.54 (3.40)		10.09 (3.25)		9.04 (3.53)		N/A		1.05 (44)		.30		
		Stress management	9.82 (1.79)		9.82 (1.79)		9.33 (2.41)		N/A		0.77 (44)		.45		
		Sleep and rest	6.45 (1.60)		6.45 (1.60)		6.71 (1.76)		N/A		−0.51 (44)		.61		
		Medication adherence and physical examination	11.52 (2.04)		11.18 (1.84)		11.83 (2.20)		N/A		−1.08 (44)		.28		
	**MetS^g^, n (%)**														
		No	21 (46)		10 (22)		11 (24)		0.0 (1)		N/A		.98		
		Yes	25 (54)		12 (26)		13 (28)		N/A		N/A		N/A		
	**MetS** **component, mean (SD)**														
		SBP^h^ (mm/Hg)	136.42 (13.01)		139.7 (11.44)		133.4 (13.83)		N/A		1.69 (44)		.10		
		DBP^i^ (mm/Hg)	83.51 (7.46)		85.18 (6.11)		81.98 (8.35)		N/A		1.47 (44)		.15		
		AC^j^ (cm)	95.73 (5.97)		94.30 (5.29)		97.05 (6.36)		N/A		−1.59 (44)		.12		
		FBS^k^ (mg/dl)	111.17 (22.22)		112.6 (27.64)		109.8 (16.27)		N/A		0.41 (44)		.68		
		HDL^l^ (mg/dl)	52.89 (13.61)		55.50 (13.32)		50.50 (13.71)		N/A		1.25 (44)		.22		
		Triglyceride (mg/dl)	114.46 (49.05)		116.5 (44.45)		112.5 (53.82)		N/A		0.27 (44)		.79		
	**Body composition, mean (SD)**														
		Body weight (kg)	73.12 (5.99)		72.53 (4.54)		73.66 (7.13)		N/A		−0.64 (44)		.52		
		Body fat mass (kg)	22.18 (4.59)		21.75 (3.51)		22.57 (5.44)		N/A		−0.61 (44)		.54		
		Body fat percent (%)	30.38 (5.31)		30.50 (4.74)		30.28 (5.88)		N/A		0.14 (44)		.89		
		Skeletal muscle mass (kg)	28.22 (3.08)		28.03 (2.96)		28.39 (3.23)		N/A		−0.39 (44)		.70		
		BMI (kg/m^2^)	25.93 (2.04)		25.65 (1.70)		26.19 (2.32)		N/A		−0.89 (44)		.38		
	**HRQoL^m^ domains, mean (SD)**														
		Urinary incontinence	67.61 (25.99)		69.17 (26.56)		66.17 (25.93)		N/A		0.39 (44)		.70		
		Urinary irritation and obstruction	88.72 (10.59)		87.78 (10.11)		89.58 (11.16)		N/A		−0.57 (44)		.57		
		Bowel problem	95.11 (9.31)		95.83 (8.33)		94.44 (10.26)		N/A		0.50 (44)		.62		
		Sexual problem	19.28 (19.54)		21.89 (19.18)		16.89 (19.97)		N/A		0.86 (44)		.39		
		Hormonal problem	85.00 (13.86)		85.00 (78.83)		85.00 (78.29)		N/A		0.00 (44)		.99		

^a^N/A: not applicable.

^b^Fisher exact test.

^c^ADT: androgen deprivation therapy.

^d^LHRH: luteinizing hormone–releasing hormone.

^e^PSA: prostate-specific antigen.

^f^Lifestyle score measured by lifestyle evaluation; scores range from 36 to 144.

^g^MetS: metabolic syndrome.

^h^SBP: systolic blood pressure.

^i^DBP: diastolic blood pressure.

^j^AC: abdominal circumference.

^k^FBS: fasting blood sugar.

^l^HDL: high-density lipoprotein.

^m^HRQoL: health-related quality of life. Scores range from 0 to 100, higher scores represent better HRQoL.

The average number of comorbidities was 1.61 (SD 1.04), and the most common comorbid diseases were cardiovascular diseases such as hypertension or dyslipidemia (31/46, 67%). There was no difference in comorbidities between the experimental and waitlist control groups (*P*=.87). Of the 65 participants, 30 (65%) received antiandrogen monotherapy, and the rest received antiandrogen therapy with luteinizing hormone–releasing hormone. The average duration of ADT was 40.63 (SD 24.71) months, the mean prostate-specific antigen level was 0.07 (SD 0.19) ng/ml, and the mean Gleason score, which determines the prognosis and pathological status of cancer, was 7.80 (SD 0.98). The experimental and waitlist control groups showed no statistical differences between general and disease-related characteristics. The mean healthy lifestyle score was 99.07 (SD 13.56). The MetS components with mean values in an abnormal range were SBP (mean 136.42, SD 13.01 mm Hg), AC (mean 95.73, SD 5.97 cm), and FBS (mean 111.17, SD 22.22 mg/dL). There were no statistical differences in MetS components between the groups. The mean body weight and BMI were approximately 73 (SD 5.99) kg and 26 (SD 2.04) kg/m^2^, respectively. The participants responded that they had problems in the urinary incontinence (mean 67.61, SD 25.99) and sexual (mean 19.28, SD 19.54) domains of HRQoL. The experimental and waitlist control groups were homogeneous in terms of their main outcome variables.

### Results of Primary Outcome Variables Between Groups Over Time

**Figure 3:**
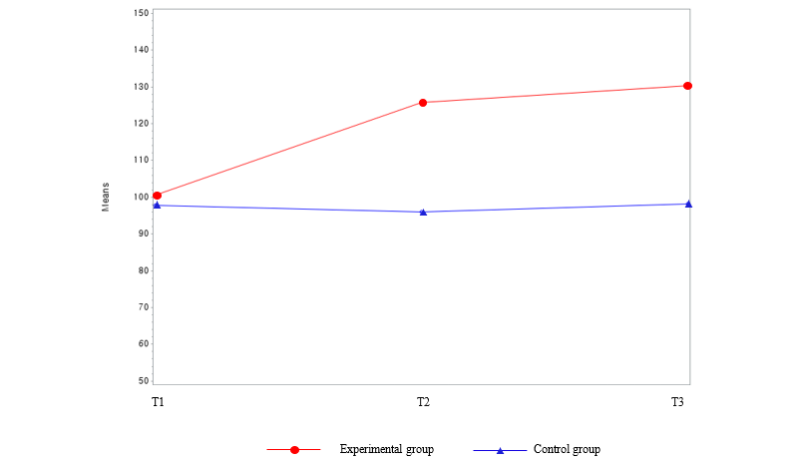
Group comparison among the main outcome variables (Lifestyle score) over time. T1: time point 1; T2: time point 2; T3: time point 3.

Table 2 and Figure 3 show the results of the lifestyle score variables between the groups over time. The study found that there were no group differences in lifestyle scores at baseline (Table 1). However, over time, the experimental group’s lifestyle scores consistently increased (T1=100.55, T2=125.82, and T3=130.27), whereas the waitlist control group’s lifestyle scores showed no consistent increase (T1=97.71, T2=95.92, and T3=98.21). Over time, the lifestyle scores of the experimental group significantly increased (β=29.23; *P*≤.001) compared with the waitlist control group. The experimental group developed a healthier lifestyle with time, and remarkable changes were observed during the intervention period.

**Table 2:** Group comparison in lifestyle score, MetS^a^ components, body composition, and HRQoL^b^ domain parameters (N=46).

Outcome and group				T1^c^, mean (SD)		T2^d^, mean (SD)		T3^e^, mean (SD)		Difference of changes between groups over time^f^				
										Estimate (SE; 95% CI)		*F* test (*df*)		*P* value
**Lifestyle score**														
	Experimental			100.55 (12.53)		125.82 (11.73)		130.27 (10.15)		29.23 (3.50; 22.26 to 36.19)		38.49 (88)		≤.001
	Control			97.71 (14.57)		95.92 (18.64)		98.21 (15.07)		29.23 (3.50; 22.26 to 36.19)		38.49 (88)		≤.001
**MetS components**														
	**SBP^g^**													
		Experimental	139.73 (11.44)		N/A^h^		126.25 (14.08)		−5.64 (3.72; −13.12 to 1.85)		2.30 (44)		.14	
		Control	133.38 (13.83)		N/A		125.54 (12.58)		−5.64 (3.72; −13.12 to 1.85)		2.30 (44)		.14	
	**DBP^i^**													
		Experimental	85.18 (6.11)		N/A		79.36 (9.35)		−0.94 (2.21; −5.40 to 3.52)		0.18 (44)		.67	
		Control	81.98 (8.35)		N/A		77.10 (7.36)		−0.94 (2.21; −5.40 to 3.52)		0.18 (44)		.67	
	**FBS^j^**													
		Experimental	112.64 (27.64)		N/A		102.59 (12.42)		−12.0 (6.02; −24.14 to 0.13)		3.98 (44)		.05	
		Control	109.83 (16.27)		N/A		111.79 (16.88)		−12.0 (6.02; −24.14 to 0.13)		3.98 (44)		.05	
	**HDL^k^ cholesterol**													
		Experimental	55.50 (13.33)		N/A		55.32 (12.46)		−0.27 (1.48; −3.25 to 2.72)		0.03 (44)		.86	
		Control	50.50 (13.71)		N/A		50.58 (12.34)		−0.27 (1.48; −3.25 to 2.72)		0.03 (44)		.86	
	**Triglyceride**													
		Experimental	116.55 (44.45)		N/A		107.73 (43.19)		−11.65 (12.04; −35.91 to 12.61)		0.94 (44)		.34	
		Control	112.54 (53.82)		N/A		115.38 (62.71)		−11.65 (12.04; −35.91 to 12.61)		0.94 (44)		.34	
	**AC^l^**													
		Experimental	94.30 (5.29)		N/A		91.02 (3.94)		−2.49 (1.23; −4.98 to −0.01)		4.09 (44)		.049	
		Control	97.05 (6.36)		N/A		96.27 (6.83)		−2.49 (1.23; −4.98 to −0.01)		4.09 (44)		.049	
**Body composition**														
	**Body weight (kg)**													
		Experimental	72.53 (4.54)		N/A		70.04 (4.53)		−1.52 (0.46; −2.45 to −0.58)		10.71 (44)		<.001	
		Control	73.66 (7.13)		N/A		72.68 (7.04)		−1.52 (0.46; −2.45 to −0.58)		10.71 (44)		<.001	
	**BMI (kg/m^2^)**													
		Experimental	25.65 (1.70)		N/A		24.76 (1.49)		−0.55 (0.17; −0.88 to −0.21)		10.54 (44)		<.001	
		Control	26.19 (2.32)		N/A		25.84 (2.27)		−0.55 (0.17; −0.88 to −0.21)		10.54 (44)		<.001	
	**Skeletal muscle mass (kg)**													
		Experimental	28.03 (2.96)		N/A		27.84 (4.19)		0.53 (0.83; −1.15 to 2.20)		0.40 (44)		.53	
		Control	28.39 (3.23)		N/A		27.67 (2.84)		0.53 (0.83; −1.15 to 2.20)		0.40 (44)		.53	
	**Fat mass (kg)**													
		Experimental	21.75 (3.51)		N/A		19.76 (5.01)		−1.93 (1.35; −4.65 to 0.78)		2.06 (44)		.16	
		Control	22.57 (5.44)		N/A		22.52 (4.66)		−1.93 (1.35; −4.65 to 0.78)		2.06 (44)		.16	
	**Fat percentage**													
		Experimental	30.50 (4.74)		N/A		28.32 (7.27)		−2.75 (1.90; −6.58 to 1.09)		2.08 (44)		.16	
		Control	30.28 (5.88)		N/A		30.84 (4.61)		−2.75 (1.90; −6.58 to 1.09)		2.08 (44)		.16	
**HRQoL domains**														
	**Urinary incontinence**													
		Experimental	69.17 (26.56)		81.19 (18.87)		83.38 (18.67)		7.70 (6.55; −5.31 to 20.72)		0.70 (88)		.50	
		Control	66.18 (25.93)		72.29 (23.00)		72.68 (29.53)		7.70 (6.55; −5.31 to 20.72)		0.70 (88)		.50	
	**Urinary irritation and obstruction**													
		Experimental	87.78 (10.11)		94.60 (7.54)		97.73 (4.93)		14.63 (4.05; 6.57 to 22.69)		7.01 (88)		<.001	
		Control	89.58 (11.16)		89.32 (11.43)		84.90 (18.14)		14.63 (4.05; 6.57 to 22.69)		7.01 (88)		<.001	
	**Bowel**													
		Experimental	95.83 (8.33)		95.64 (7.21)		98.48 (4.37)		2.30 (2.98; −3.61 to 8.22)		0.34 (88)		.71	
		Control	94.44 (10.26)		91.67 (14.33)		94.79 (10.15)		2.30 (2.98; −3.61 to 8.22)		0.34 (88)		.71	
	**Sexual**													
		Experimental	21.89 (19.18)		18.86 (16.02)		25.49 (16.71)		1.52 (4.50; −7.43 to 10.47)		0.08 (88)		.92	
		Control	16.89 (19.97)		12.15 (12.18)		18.97 (15.03)		1.52 (4.50; −7.43 to 10.47)		0.08 (88)		.92	
	**Hormonal**													
		Experimental	85.00 (11.65)		89.32 (12.94)		92.73 (7.03)		6.27 (3.48; −0.64 to 13.18)		1.85 (88)		.16	
		Control	85.00 (15.88)		86.88 (12.58)		86.46 (13.87)		6.27 (3.48; −0.64 to 13.18)		1.85 (88)		.16	

^a^MetS: metabolic syndrome.

^b^HRQoL: health-related quality of life.

^c^T1: time point 1.

^d^T2: time point 2.

^e^T3: time point 3.

^f^Reference: interaction between control group and T1.

^g^SBP: systolic blood pressure.

^h^N/A: not applicable.

^i^DBP: diastolic blood pressure.

^j^FBS: fasting blood sugar.

^k^HDL cholesterol: high-density lipoprotein cholesterol.

^l^AC: abdominal circumference.

### Results of Secondary Outcome Variables Between Groups Over Time

**Figure 4:**
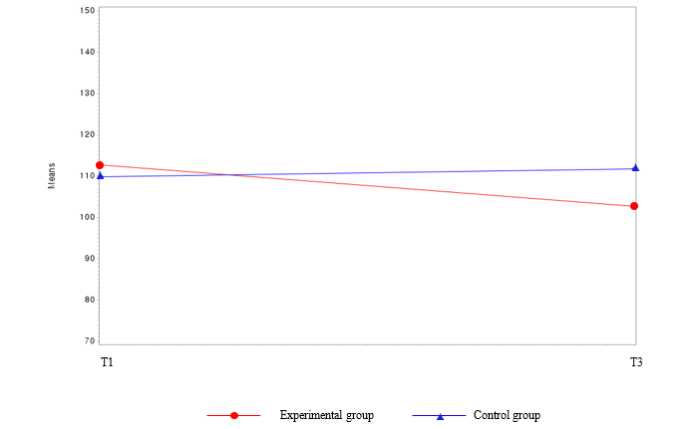
Group comparison among the main outcome variables (fasting blood sugar) over time. T1: time point 1; T3: time point 3.

**Figure 5:**
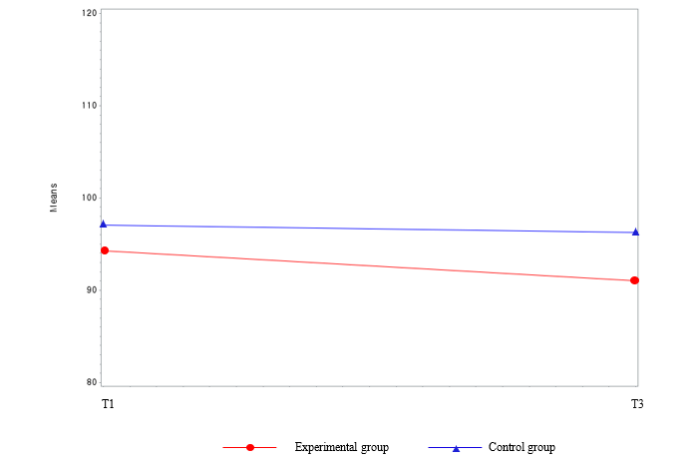
Group comparison among the main outcome variables (abdominal circumference) over time. T1: time point 1; T3: time point 3.

**Figure 6:**
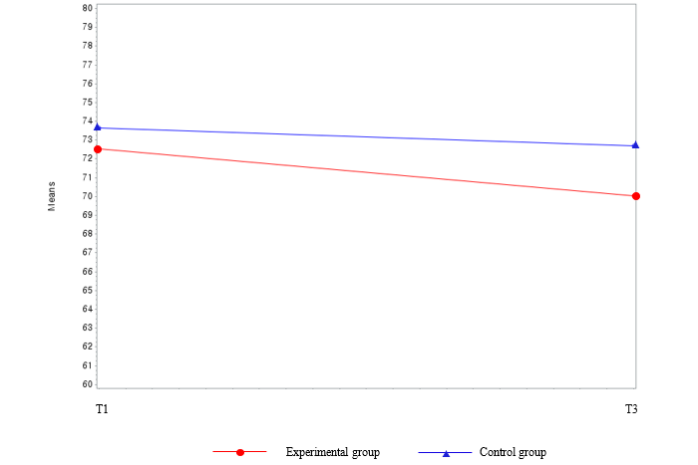
Group comparison among the main outcome variables (body weight) over time. T1: time point 1; T3: time point 3.

**Figure 7:**
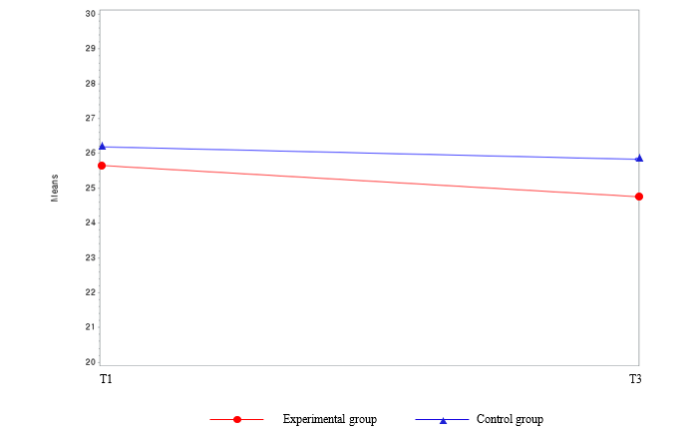
Group comparison among the main outcome variables (BMI) over time. T1: time point 1; T3: time point 3.

**Figure 8:**
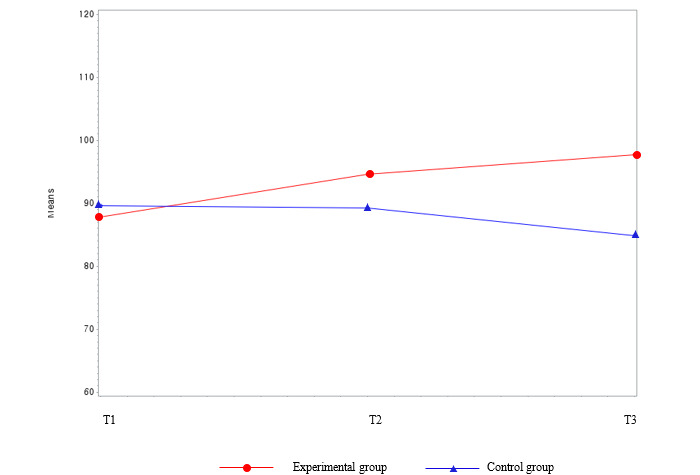
Group comparison among the main outcome variables (urinary irritation and obstruction) over time. T1: time point 1; T2; time point 2; T3: time point 3.

The difference in the prevalence of MetS before and after the intervention between the experimental group and the waitlist control group was not statistically significant (*χ*^2^_1_=1.1; *P*=.31 at T3 [not presented in the tables]). Among the MetS components, the parameters for FBS (β=−12.0; *F*_44_=3.98; *P*=.05) and AC (β=−2.49; *F*_44_=4.09; *P*=.049) showed significant interactions between group and time (Table 2 and Figures 4 and 5). Regarding body composition, the mean body weight and BMI in the experimental group decreased significantly by 1.52 kg and 0.55 kg/m^2^, respectively (*P*<.001) compared with the baseline values. Group, time, and group and time interactions were also statistically significant between these variables (*P*<.001). Over time, the mean body weight (*P*<.001) and BMI (*P*<.001) decreased more in the experimental group than in the waitlist control group (Table 2 and Figures 6 and 7). Regarding HRQoL domains (Table 2 and Figure 8), a more significant improvement was observed in the experimental group than in the waitlist control group for the urinary irritative and obstructive domain. The mean changes in the urinary irritative and obstructive domain of HRQoL were statistically significant at each time point from the baseline, whereas the mean changes in the control group for this domain were not statistically significant. Group and time interactions were not significant, except in the urinary irritative and obstructive domain of HRQoL in the experimental group (β=14.63; *F*_8,8_=7.01; *P*<.001).

## Discussion

### Principal Findings

This study was conducted to determine the effectiveness of a nurse-led mobile-based education program for patients with PC who are at risk of MetS. This study showed that a nurse-led mobile-based health coaching program promoted a healthy lifestyle in patients with PC receiving ADT, which ultimately improved the levels of some MetS components (eg, reduction in FBS and AC), body composition (eg, reduction in weight and BMI), and HRQoL (eg, the urinary irritative and obstructive domain).

In this study, with a 3-month intervention, the results indicated that each variable required a different amount of time to show changes. In a previous study, a period of at least 3 to 8 weeks was required to confirm significant changes in weight, BMI, and the levels of each MetS component following lifestyle intervention programs [48-50]. Specifically, AC, FBS, body weight, and BMI have been found to decrease significantly over short periods [49,51]. However, in studies with intervention periods of ≥6 months, significant results were confirmed for MetS components including HDL cholesterol, SBP, and DBP [52,53]. Even in weight control programs that included strict diet control and exercise intervention guidelines, MetS components, including HDL cholesterol, did not change over a period of 8 weeks and only showed a significant change in both men and women 10 months after the end of the intervention [54].

The study by Dawson et al [55] observed decreases in body weight and AC but not in HDL cholesterol level among the MetS components in patients with PC. Focht and colleagues [56] also reported decreased bodyweight in patients with PC. These studies included lifestyle interventions lasting 3 months for patients with PC who had received ADT. The mean duration of ADT was 14 months in the study by Focht et al [56] and 30 months in the study by Dawson et al [55]. Specifically, Focht et al [56] confirmed that the body composition of body weight and body fat decreased significantly at 3 months compared with the control group. Furthermore, Dawson et al [55] conducted a program that emphasized exercise and protein supplementation for 3 months in patients with PC receiving ADT. As a result, of the MetS components, the AC of participants decreased most significantly in the experimental group than in the waitlist control group.

Reductions in body weight, AC, and FBS levels were closely related. When beginning to lose weight, the body temporarily lowers its metabolic function to maintain homeostasis and first metabolizes glucose, which is a basic energy source. When stored glycogen is broken down, the insulin mechanism is activated for additional energy consumption [57]. Weight loss causes a decrease in FBS level along with the action of insulin, which reactivates FBS, stored in the form of excess fat in the liver or abdomen. This fat is continuously used to generate energy, consequently, the fat accumulated in the liver or abdomen is consumed, leading to a reduced volume [58]. In this study, although no significant decrease in body fat was observed, a decrease in AC was observed. Similarly, in previous studies, decreases in body weight [56], AC [55], and FBS levels [49] were confirmed 3 months after the intervention.

In this study, there were no significant changes in body composition related to body fat and skeletal muscle mass or in MetS components related to blood pressure (SBP and DBP) and lipids (HDL cholesterol and triglyceride). Insulin resistance develops over a long period, which increases the risk of obesity, diabetes, and MetS. In addition, it affects the lipid ratio, leading to an increase in low-density lipoprotein cholesterol and a decrease in HDL cholesterol [58], and causes inflammatory changes, resulting in changes in blood pressure owing to an increase in the residual amount of sodium in the blood [59]. Moreover, in a study that examined prediabetic patients diagnosed with impaired fasting glucose levels over the course of a 10-year follow-up period, 37% of the patients developed diabetes [60]. This finding indicates that the disease mechanism does not change over a short period but rather progresses slowly. Therefore, a long-term follow-up study is required to more accurately confirm the effectiveness of a lifestyle intervention program for MetS.

Among the HRQoL domains, an effect was observed only in the urinary irritative and obstructive domain, which may be caused by the education on how to manage the side effects of ADT treatment in the third and fourth weeks of the intervention. Patients with PC who have undergone multiple surgeries or frequent radiation therapy complained of side effects [61], such as urinary irritation and frequency. During the intervention in this study, information about appropriate water intake and pelvic floor muscle exercises to relieve urinary irritative and obstructive symptoms such as urinary irritation and frequency were included in an educational brochure [57], and appropriate water intake and pelvic floor muscle exercise were recommended for the participants depending on the presence or absence of symptoms. Thus, participants with these symptoms may have experienced relief through this intervention. Of the remaining HRQoL domains, the urinary incontinence, bowel, sexual, and hormonal domains did not show statistically significant changes. HRQoL was assessed using a questionnaire on treatment side effects and symptoms during the preceding 4 weeks. Most participants in this study were receiving long-term treatment after surgery, with an average of 40 (SD 24.71) months of ADT treatment. The bowel domain contains questions about whether the participants had diarrhea and bloody stools, which are acute side effects of participants who have undergone PC resection surgery. This domain had a higher mean score than the other HRQoL domains, with an average of ≥95 beginning at the baseline; therefore, these questions might not be relevant for participants of this study who were not in the acute stage after surgery. In addition, most questions in the hormonal domain examined the side effects that appear toward the start of ADT such as breast tenderness, bloody stools, and weight loss. Therefore, the insignificant changes in this domain might be because the participants in this study had already been taking medication for these side effects. At 19 points, the sexual domain had the lowest mean score of all the HRQoL domains. Sexual function might be limited in recovery as a result of a short-term health coaching program [62,63]. Although a previous study found that exercise can improve sexual function [64], sexual function requires interaction with a partner [65]. To restore sexual function, it is necessary to combine both psychological intervention and drug treatment [66].

This study found lifestyle changes to be critical in reducing the risk of MetS and that improved exercise and nutritional regimens should be implemented consistently for at least 3 months. Furthermore, a patient-centered, individualized approach that considers the side effects of ADT is needed to increase adherence.

### Limitations

This study had some limitations. First, the criteria for participant selection included patients with at least 1 MetS component. To more thoroughly examine the effectiveness of this program on MetS management, stricter inclusion criteria should have been applied, such as only including those who were being treated with luteinizing hormone–releasing hormone, which is a type of ADT that causes the occurrence of more MetS components MetS components; those with ≥3 MetS components; those with a sedentary lifestyle; or those with a specific time after radical prostatectomy. Owing to data unavailability, this study only collected the Gleason score but not the information on TNM classification as the patient’s pathological data. It is important to establish accurate patient pathological criteria; hence, future studies should incorporate both the TNM classification and the Gleason score with accurate criteria. Second, the 3-month intervention period planned in this study was limited to evaluating the continuation of self-management and improvement in MetS risk factors. It is necessary to extend the intervention period to at least 6 months to further evaluate the persistence of self-management. As older men with PC receiving ADT are at high risk of osteoporosis, measuring bone health–related indicators in addition to changes in body composition is highly recommended for future studies. Third, it is difficult to generalize the results of this study because the participants were recruited from a single hospital and not from a multicenter. Fourth, obtaining low Cronbach α values for some items in the lifestyle score may indicate a mismatch between the measurement and the participant's lifestyle. This is because the lifestyle score was created for the general adult population; however, the participants in this study were older adults who had PC, had undergone surgery, and were taking hormone-suppressing medications.

### Conclusions

A nurse-led mobile-based health coaching program was developed to promote a healthy lifestyle among patients with PC receiving ADT. The research findings confirmed that lifestyle changes through nurse-led education can improve the components of MetS, body composition, and ADT’s side effects. Therefore, by participating in this program, nurses were capable of creating changes in patients’ lifestyles and improving the self-management of patients who were beginning ADT for the first time. In addition, this study can provide a basis for the development of other mobile-based education programs and tools.

## Appendix

Multimedia Appendix 1 CONSORT-EHEALTH checklist (V 1.6.1).

## Abbreviations

AC: abdominal circumference
ADT: androgen deprivation therapy
DBP: diastolic blood pressure
EMR: electronic medical record
EPIC: expanded prostate cancer index composite
EPIC-26: 26-item Expanded Prostate Cancer Index Composite
FBS: fasting blood sugar
HDL: high-density lipoprotein
HRQoL: health-related quality of life
IMB: information-motivation-behavioral
MetS: metabolic syndrome
PC: prostate cancer
SBP: systolic blood pressure
T1: time point 1
T2: time point 2
T3: time point 3
